# Anesthesia Management Involving Remimazolam in a Patient With Pulmonary Hypertension and Right Ventricular Failure: A Case Report

**DOI:** 10.7759/cureus.86568

**Published:** 2025-06-22

**Authors:** Masato Ryo, Yusuke Ishida, Masahiro Hamaguchi, Maiko Hosokawa, Katsunori Oe

**Affiliations:** 1 Department of Anesthesiology, St. Luke's International Hospital, Tokyo, JPN; 2 Anesthesiology, Showa Medical University Hospital, Tokyo, JPN

**Keywords:** cardiac failure, percutaneous left atrium appendage closure, pulmonary hypertension, pulmonary vascular resistance (pvr), remimazolam, right ventricular dysfunction

## Abstract

In the anesthetic management of patients with pulmonary hypertension (PH), it is essential to minimize right ventricular (RV) load and prevent abrupt changes in pulmonary vascular resistance (PVR). We report a case in which safe anesthetic management was achieved without circulatory collapse by using remimazolam in a patient with PH. An 84-year-old woman was scheduled to undergo percutaneous left atrial appendage closure for non-valvular atrial fibrillation. She had developed secondary PH and RV failure secondary to atrial fibrillation and systemic sclerosis, necessitating careful general anesthesia with positive pressure ventilation. Total intravenous anesthesia was administered using remifentanil and remimazolam. Hemodynamic stability was maintained throughout induction and intraoperative management. Transesophageal echocardiography performed during the procedure revealed no exacerbation of PH or RV failure, and the percutaneous catheter procedure was completed uneventfully. The patient’s postoperative course was also uneventful, and she was discharged without any serious complications. Anesthesia management using remimazolam might be beneficial in patients with PH, as it provides stable hemodynamics and allows for safe perioperative care.

## Introduction

In the anesthetic management of patients with pulmonary hypertension (PH), it is crucial to minimize right ventricular (RV) load and avoid sudden fluctuations in pulmonary vascular resistance (PVR). In Japan, remimazolam, a short-acting benzodiazepine, became available for use during general anesthesia in 2020. This agent has been shown to have minimal depressive effects on cardiovascular parameters, such as cardiac output and systemic blood pressure [1,2], and is also expected to exert limited influence on PVR. However, there are few reports on the use of remimazolam for PH and RV failure. Here, we report a case in which general anesthesia for percutaneous left atrial appendage closure (LAAC) in a patient with PH and RV failure was safely managed using remimazolam. Written informed consent was obtained from the patient for the publication of this case report.

## Case presentation

**Figure 1:**
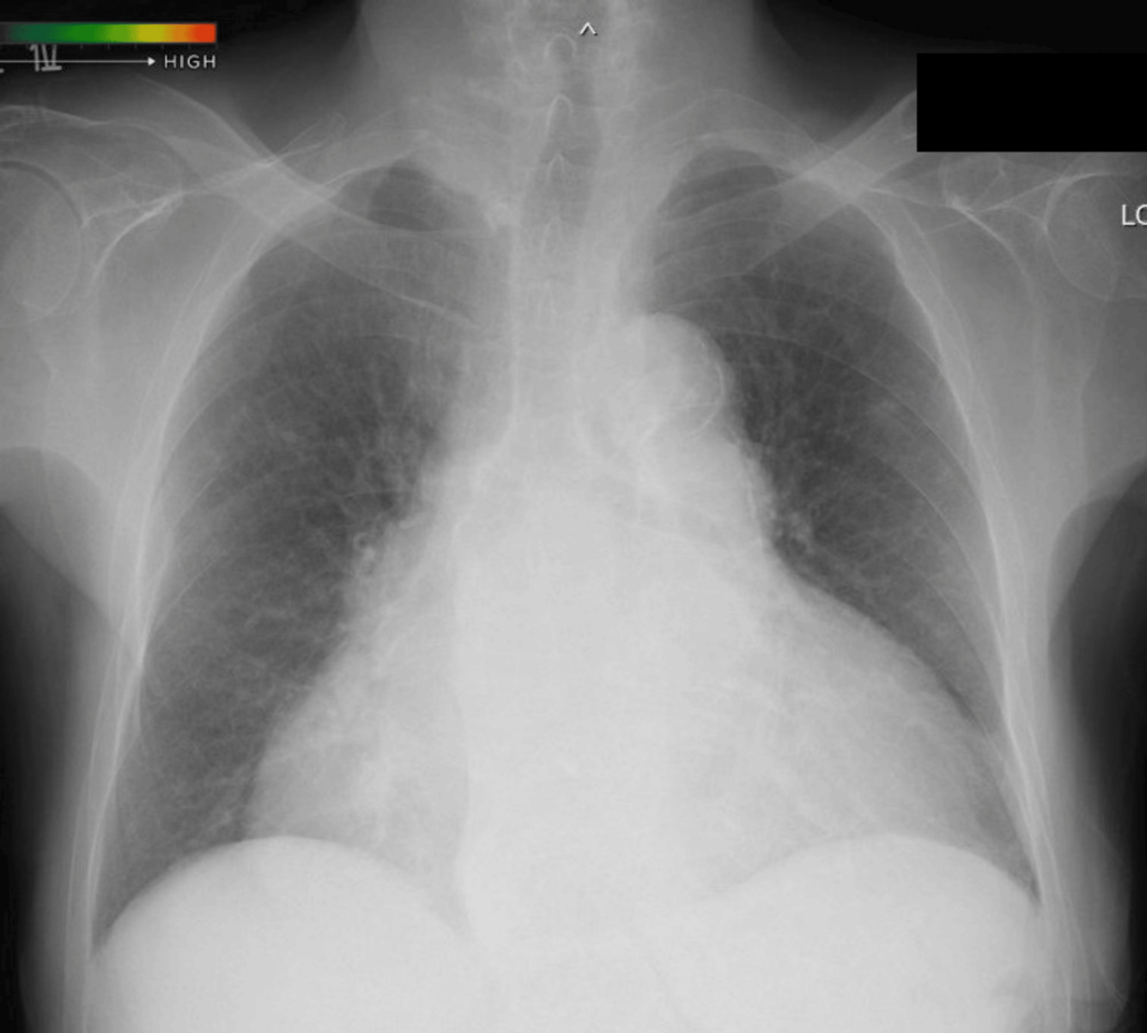
Preoperative chest X-ray Significant cardiac enlargement and a cardiothoracic ratio of 77.4% were noted.

**Figure 2:**
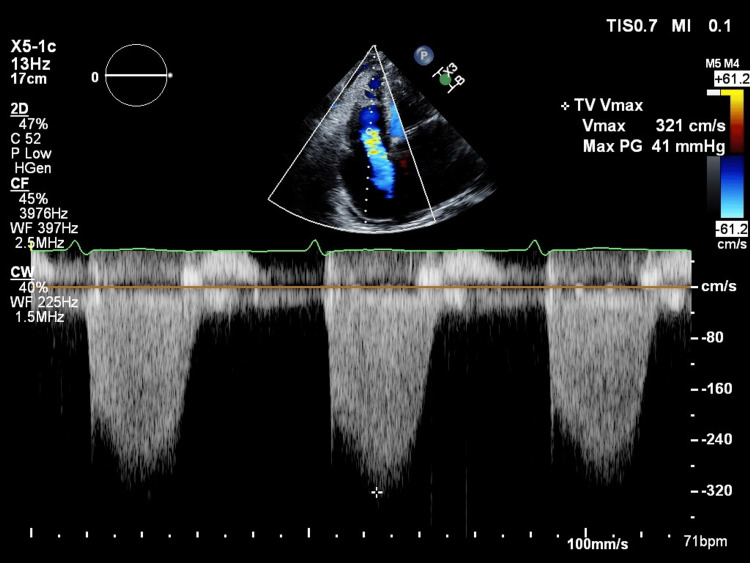
Preoperative transthoracic echocardiography findings Atrial fibrillation and secondary pulmonary hypertension were present, and echocardiography revealed findings suggestive of moderate tricuspid regurgitation.

The patient was an 84-year-old woman, 146 cm tall, weighing 52.9 kg, with a history of persistent non-valvular atrial fibrillation, for which she had been taking edoxaban for anticoagulation. Two months prior to the surgery described here, she developed gastrointestinal bleeding. Further investigation revealed an arteriovenous malformation (AVM) in the transverse colon as the source of bleeding. Since the AVM was deemed difficult to treat definitively and continued edoxaban use could have exacerbated gastrointestinal bleeding, it was discontinued, resulting in the improvement of the bleeding. Based on this clinical course, continuation of anticoagulation therapy was considered inappropriate, and percutaneous LAAC was planned as an alternative stroke prevention strategy. The patient also had PH secondary to systemic sclerosis, for which she was receiving treatment with a phosphodiesterase type five inhibitor, an endothelin receptor antagonist, and a prostaglandin I₂ analog. Complications of PH included Child-Pugh class B liver cirrhosis (seven points; total bilirubin and PT-INR are three points; the other three factors are one point) and RV failure. Chest X-ray revealed marked cardiomegaly with a cardiothoracic ratio of 77.4% (Figure 1), and her New York Heart Association classification was class IV. Preoperative transthoracic echocardiography showed preserved left ventricular systolic function (left ventricular ejection fraction: 61%); however, there was significant left atrial enlargement due to atrial fibrillation (long diameter × short diameter: 83 × 62 mm; left atrial volume index: LAVI = 122 mL/m²). Her RV function was impaired, with an RV S’ of 9.2 cm/s, a tricuspid annular plane systolic excursion of 10 mm, and an elevated tricuspid regurgitation pressure gradient (TRPG) of 41 mmHg (Figure 2), findings consistent with PH and RV failure.

**Figure 3:**
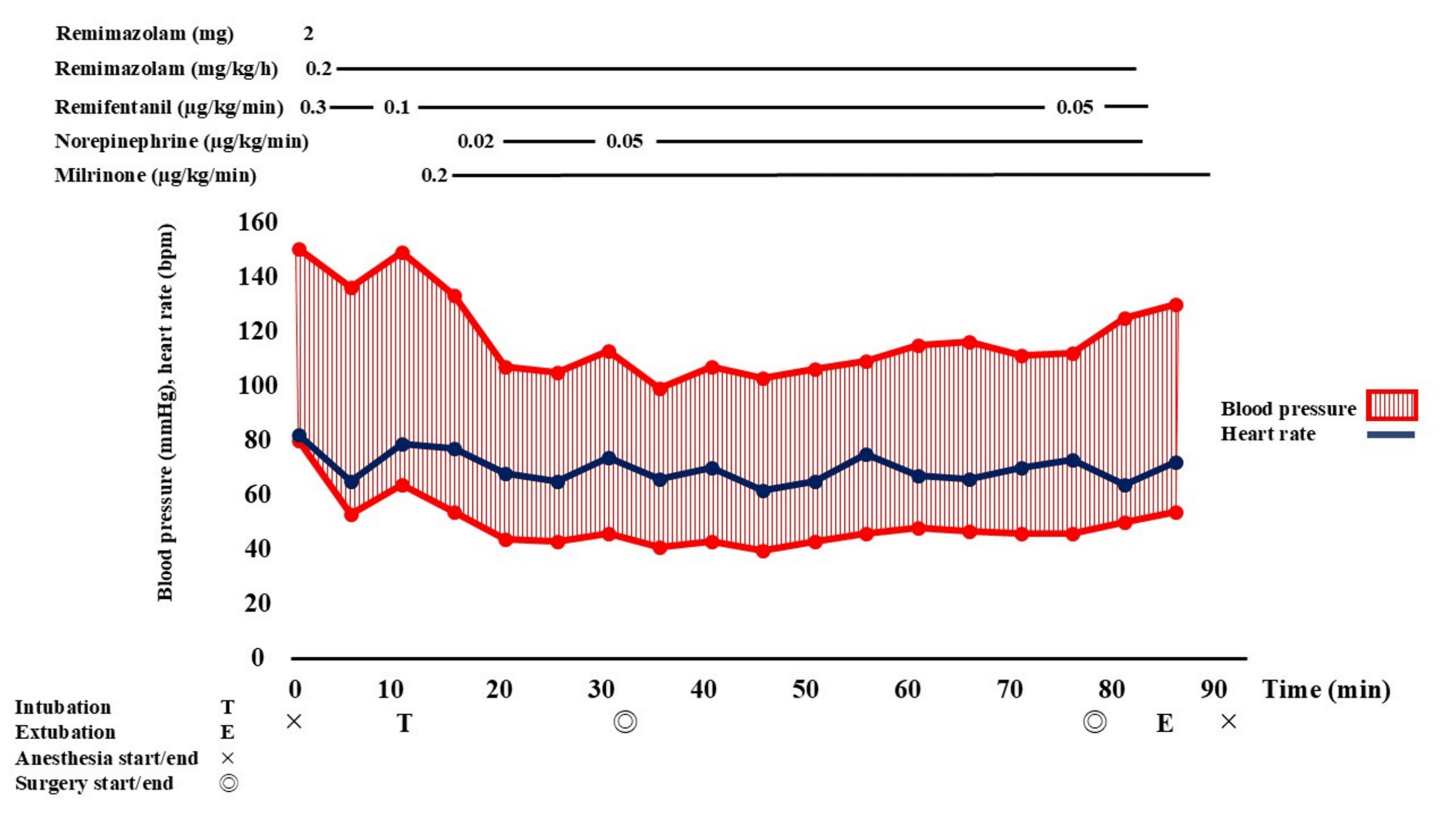
Patient’s anesthesia record Cardiovascular dynamics remained stable during the management of anesthesia with remimazolam.

Due to the risk of worsening PH with intraoperative positive pressure ventilation, a careful anesthetic strategy was essential to avoid increases in PVR and circulatory collapse. Hence, general anesthesia (ASA-PS 3) was planned with total intravenous anesthesia (TIVA) using remimazolam and remifentanil, with inhaled nitric oxide (iNO) preinstalled in the ventilator circuit as a precaution. Anesthesia induction was initiated with a 2 mg bolus of remimazolam, followed by its continuous infusion at 0.2 mg/kg/h. Remifentanil was administered at 0.3 µg/kg/min, and rocuronium 50 mg was used for muscle relaxation. A 7.0 mm endotracheal tube was inserted, during which only a mild decrease in blood pressure was observed. Anesthesia was maintained with a remimazolam infusion of 0.2 mg/kg/h and remifentanil at 0.1 µg/kg/min. Milrinone at 0.2 µg/kg/min was used for inotropic support, and hypotension was managed with a continuous infusion of norepinephrine at 0.02-0.05 µg/kg/min (Figure 3). Intraoperative monitoring included invasive arterial pressure, SpO₂, Patient State Index (PSI), and transesophageal echocardiography (TEE). According to our institutional policy, developed in consensus with the cardiology department, neither central venous nor pulmonary artery catheters were used for the percutaneous LAAC procedure, and noninvasive hemodynamic monitoring devices were not employed. The TEE probe was inserted after induction and used intraoperatively to assess the status of PH and RV function in conjunction with the LAAC procedure. Mechanical ventilation was managed with pressure-controlled ventilation, a positive end-expiratory pressure of 5 cm H₂O, and a tidal volume of 6-8 mL/kg, with an FiO₂ of 50%. Inhaled iNO was ultimately not required during the percutaneous catheter procedure. Extubation was performed under a continuous infusion of remifentanil at 0.05 µg/kg/min following administration of flumazenil 0.2 mg, in an effort to prevent RV overload associated with bucking during emergence. Our anesthetic approach resulted in no worsening of the patient’s PH and RV failure, and no significant circulatory compromise occurred from induction to emergence. The anesthesia time was one hour and 32 minutes, surgical duration was 44 minutes, total fluid intake was 520 mL, urine output was 200 mL, and intraoperative blood loss was minimal. On postoperative day one, no major anesthesia-related complications, such as intraoperative awareness, were noted. Regarding circulatory status, there were no serious complications, such as heart failure, and the patient was discharged in good condition on postoperative day 10.

## Discussion

PH is a heterogeneous clinical condition primarily characterized by an abnormal elevation in pulmonary arterial pressure. The resultant pulmonary vascular changes involve pathological remodeling and vasoconstriction of the pulmonary arteries and veins, leading to progressive dyspnea, exercise intolerance, RV failure, and ultimately death. Perioperative management of patients with PH requires careful attention to the risks of RV failure, arrhythmia, myocardial ischemia, and refractory hypoxemia [3,4]. Therefore, it is critical to preoperatively evaluate the choice of anesthesia technique and anesthetic agents, as well as strategies for preventing and managing PH. Adequate sedation and analgesia are essential to avoid exacerbation of PH during anesthesia. In addition, it is important to prevent fluid overload, hypoxemia, hypercapnia, hypothermia, and atelectasis [5,6]. Patients with PH often develop RV failure due to prolonged RV overload. While excessive fluid administration during anesthesia can worsen RV failure, insufficient fluid replacement might lead to circulatory collapse when anesthetics are administered. Thus, careful fluid management is also necessary [7].

When managing anesthesia in patients with PH, anesthetic induction should be performed with caution to avoid hemodynamic collapse. Furthermore, since agitation during emergence may also lead to increased PVR and subsequent RV overload, we attempted to prevent this under a low-dose continuous infusion of remifentanil during extubation. A key goal is to minimize increases in PVR, requiring careful selection and dosing of sedatives and analgesics. Although inhalational anesthetics, such as sevoflurane, might have limited effects on lowering PVR, they might depress RV function and should therefore be used with caution [8]. Remimazolam, an intravenous anesthetic agent approved for use in Japan in 2020, is a short-acting benzodiazepine with a shorter half-life compared to conventional benzodiazepines, and has a specific antagonist available [9]. Compared to propofol, a commonly used sedative in TIVA, remimazolam exerts milder effects on hemodynamics [10]. In addition, several reports suggest that it has a minimal impact on PVR [11,12]. These characteristics suggest that remimazolam allows for anesthetic management with minimal circulatory depression and without elevating PVR. The use of agents such as milrinone and iNO might also be considered to prevent increases in PVR due to various factors, such as surgical stress [13]. In the present case, preparation for the potential use of these agents is considered to have contributed to the stable anesthetic management without deterioration in hemodynamics. In this case, TEE was used intraoperatively to continuously monitor PH and RV function, and no significant changes were observed compared to the preoperative state.

Regarding remimazolam dosing during anesthesia, maintaining an appropriate infusion rate is crucial. Therefore, intraoperative adjustment using brain function monitoring, such as Sedline (Masimo, Irvine, CA, USA), is desirable [14]. In this case, although the remimazolam infusion rate of 0.2 mg/kg/h appeared somewhat low, anesthetic depth was managed while monitoring the PSI, which remained between 30 and 50 throughout surgery. No intraoperative awareness or related complications were observed. Elderly patients reportedly have increased sensitivity to remimazolam [15,16]. In the present case, in addition to this background factor, the patient also had impaired cardiac function, which might have allowed for anesthetic management with a low dose of remimazolam. In patients with PH and RV failure, meticulous respiratory and hemodynamic management is essential to prevent circulatory collapse. Therefore, anesthetic strategies aimed at minimizing increases in PVR, as described above, must always be considered. Our experience in the present case suggests that remimazolam might be useful for minimizing fluctuations in PVR. Moving forward, the use of remimazolam should be more actively considered in the anesthetic management of patients with PH. While knowledge on the relationship between remimazolam and systemic vascular resistance or cardiac output has increased, reports concerning its effect on PVR remain scarce. Further accumulation of evidence regarding the relationship between remimazolam and PVR is warranted.

## Conclusions

We experienced a case of LAAC in a patient with PH and RV failure in whom anesthetic management was performed involving remimazolam. The use of sedatives such as propofol or inhalational anesthetics such as sevoflurane may lead to hemodynamic collapse due to their myocardial depressant effects. However, in this case, the use of remimazolam may have contributed to stable anesthetic management. Our experience suggests that remimazolam might be a useful agent for anesthesia in patients with PH. We hope that this case report will serve as one piece of evidence supporting the effectiveness of remimazolam for anesthesia management in patients with PH. Further studies are warranted to elucidate the relationship between remimazolam and PVR.
